# Combined Transcriptome and Metabolome analysis of Pitaya fruit unveiled the mechanisms underlying Peel and pulp color formation

**DOI:** 10.1186/s12864-020-07133-5

**Published:** 2020-10-22

**Authors:** Zhaoxi Zhou, Hongmao Gao, Jianhong Ming, Zheli Ding, Xing’e Lin, Rulin Zhan

**Affiliations:** Haikou Experimental Station, Chinese Academy of Tropical Agricultural Sciences (CATAS), Haikou, China

**Keywords:** Anthocyanin, Betalain, Carotenoid, Flavonoids, Fruit flesh color, Fruit skin, Metabolites

## Abstract

**Background:**

Elucidating the candidate genes and key metabolites responsible for pulp and peel coloration is essential for breeding pitaya fruit with new and improved appeal and high nutritional value. Here, we used transcriptome (RNA-Seq) and metabolome analysis (UPLC-MS/MS) to identify structural and regulatory genes and key metabolites associated with peel and pulp colors in three pitaya fruit types belonging to two different *Hylocereus* species.

**Result:**

Our combined transcriptome and metabolome analyses suggest that the main strategy for obtaining red color is to increase tyrosine content for downstream steps in the betalain pathway. The upregulation of *CYP76ADs* is proposed as the color-breaking step leading to red or colorless pulp under the regulation by *WRKY44* transcription factor. Supported by the differential accumulation of anthocyanin metabolites in red pulped pitaya fruit, our results showed the regulation of anthocyanin biosynthesis pathway in addition to betalain biosynthesis. However, no color-breaking step for the development of anthocyanins in red pulp was observed and no biosynthesis of anthocyanins in white pulp was found. Together, we propose that red pitaya pulp color is under the strict regulation of *CYP76ADs* by WRKYs and the anthocyanin coexistence with betalains is unneglectable. We ruled out the possibility of yellow peel color formation due to anthocyanins because of no differential regulation of chalcone synthase genes between yellow and green and no detection of naringenin chalcone in the metabolome. Similarly, the no differential regulation of key genes in the carotenoid pathway controlling yellow pigments proposed that the carotenoid pathway is not involved in yellow peel color formation.

**Conclusions:**

Together, our results propose several candidate genes and metabolites controlling a single horticultural attribute i.e. color formation for further functional characterization. This study presents useful genomic resources and information for breeding pitaya fruit with commercially attractive peel and pulp colors. These findings will greatly complement the existing knowledge on the biosynthesis of natural pigments for their applications in food and health industry.

## Background

Pitayas (or dragon fruit), originated from Latin America and belongs to the genus *Hylocereus* of the Cactaceae family. This fruit has gained popularity in many countries by virtue of its exotic appearance and high nutritional value. It is grown as a fruit crop worldwide mainly in tropical and subtropical regions. Pitayas have the ability to tolerate drought stress and can grow in less fertile soils and therefore can boost the economy of those areas [1]. In addition, pitayas are also a rich source of nutrients and antioxidants like vitamin C, organic acids, and pigments (flavonoids and betalains) [2, 3].

Genus *Hylocereus* consists of 14 different species and the most common ones are *Hylocereus undatus*, *Hylocereus megalanthus*, and *Hylocereus costaricensis*. Their classification is based on the color of the peel and pulp which is contributed mainly by the pigment betalains and other secondary metabolites such as anthocyanins and carotenoids [4]. Pitayas have three different peel colors (green, red, and yellow) and two types of pulp color (white and red). Red pulp pitaya has more popularity among consumers for its striking color and high nutritional value as compared to white pulp pitayas [5]. Anthocyanins are phenolic compounds that give red to purple colors to plants and thus play role in attracting pollinators [6]. Besides, they have health-promoting properties, including antioxidation, anti-mutation, prevention of cardiovascular disease, liver protection, and inhibiting the metastasis of tumor cells [7–10]. Betalains are nitrogenous-based water-soluble pigments derived from tyrosine which are known to be involved in red pulp color of pitaya fruits [11]. In addition to giving red color to fruits, betalains are considered as important fruit antioxidants that prevent the consumers from oxidative stress and several degenerative diseases [12]. It is believed that betalains color is more stable than that of anthocyanins because it does not depend on pH [13]. Betalains are also considered to play a role in plant defense against different biotic and abiotic stresses [14–17]. Carotenoids are important class of pigments under the subclass isoprenoids and are found ubiquitously in plants and micro-organisms [6]. Carotenoids give yellow to red color to fruits and have scope to serve as natural colorants in food industry [6, 18]. The biosynthetic pathway of anthocyanins is well studied in different plants and is regulated by several key genes and regulatory factors [19, 20]. However, very few studies were conducted to study the betalains biosynthesis pathway and their regulatory genes because they are present only in single plant order, namely, Caryophyllales [6].

Recently, the relatively low cost of Illumina sequencing has made it easier to perform global transcriptome profiling in any plant species. This helps us to understand the molecular mechanisms and key genes involved in defining key agronomic and quality traits [5, 21–23]. For example, tyrosine hydroxylase CYP76AD1 and 4,5-DOPA dioxygenase DODA, related to betalain biosynthesis, were identified as the key genes controlling the red pulp color of pitaya using the transcriptome approach [5]. Non-targeted metabolome profiling is also a robust technique to understand the correlation between phenotypes and their metabolites [3, 24]. It can be used to understand the metabolic composition of a particular organ or tissue and assist in breeding for improved quality. Recently, metabolome profiling of pitaya demonstrated that the decrease of amino acid, accompanied by the increase of sugars and organic acid, might contribute to the formation of betalains [3]. Although few studies performed transcriptome or metabolome profiling of pitayas with either peel or pulp, none of the studies performed a combined transcriptome and metabolome analysis of both peel and pulp tissues of pitaya fruits to have a better understanding of key genes and metabolites involved in the peel and pulp color formation [2, 3, 5]. A detailed examination of the expression and accumulation of the key genes and metabolites underlying color formation in pitayas will facilitate the breeding of colorful cultivars with improved fruit appeal. In this study, we have investigated the differentially regulated genes (DEGs) and metabolic composition of peel and pulp tissues from different colored pitayas. Our study discussed the differential expression of key genes in betalain, carotenoids, and anthocyanin biosynthesis pathways for their possible involvement in color formation in pitaya fruits.

## Results

### Analysis of transcriptome dataset and unigene assembly

**Fig. 1:**
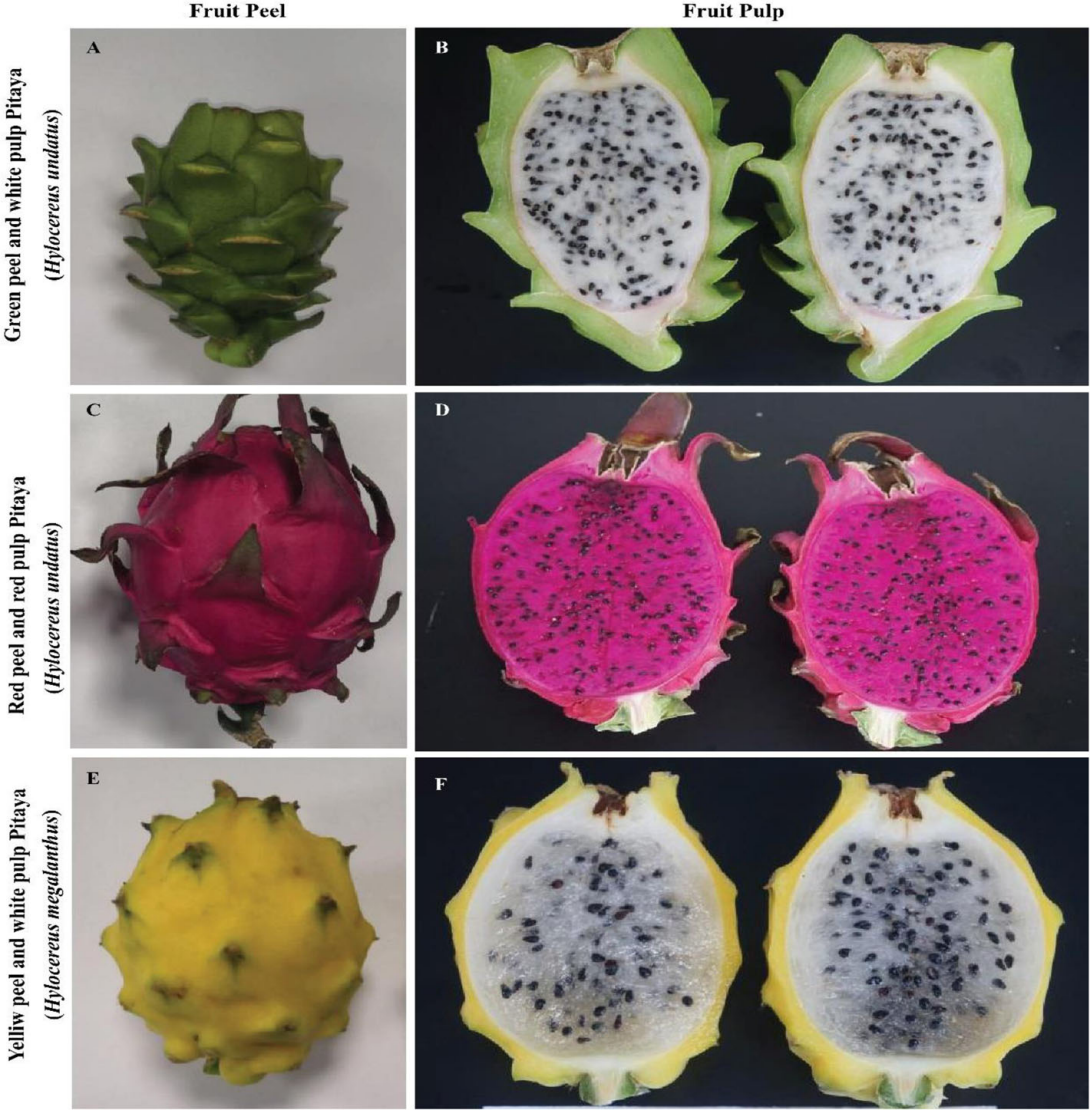
Phenotypic comparison of peel and pulp of *H. undatus*; green peel and white pulp (GW) and red peel and red pulp (RR), and *H. megalanthus*; yellow peel and white pulp (YW)

We performed a transcriptome analysis of peel and pulp tissues of three different pitaya fruits to explore potential target genes involved in pitaya fruit color (Fig. 1). The total raw and clean reads in each sample ranged from 46,688,418 to 62,038,332 and 45,033,096 to 60,311,044, respectively (Table 1). A total of 141.13 Gb clean data was obtained. The Q30% was higher than 93% and GC contents were on average 50% (Table 1) which indicates that the transcriptome results are good enough to proceed to downstream analysis.

**Table 1:** Overview of the transcriptome sequencing dataset and quality check

Samples	Raw Reads	Clean Reads	Q30 (%)	GC Content (%)
GW-peel-1	55,701,582	54,624,648	94.5	50.89
GW-peel-2	51,972,514	50,955,940	93.74	50.69
GW-peel-3	52,503,168	51,336,002	94.33	50.63
GW-pulp-1	52,995,520	49,937,440	94.62	50.49
GW-pulp-2	60,780,458	56,622,586	94.41	50.93
GW-pulp-3	57,923,082	55,294,992	94.48	50.73
RR-peel-1	46,688,418	45,033,096	93.89	48.47
RR-peel-2	57,849,830	56,017,310	93.62	48.91
RR-peel-3	54,541,504	52,646,804	94.25	48.61
RR-pulp-1	50,555,020	48,714,322	94.1	49.43
RR-pulp-2	50,648,636	49,387,298	93.46	49.05
RR-pulp-3	53,741,688	52,386,128	93.92	49.61
YW-peel-1	55,656,600	53,925,590	94.08	50.58
YW-peel-2	53,519,622	51,785,646	94.07	50.57
YW-peel-3	58,784,674	57,361,112	94.54	50.42
YW-pulp-1	62,038,332	60,311,044	93.8	50.29
YW-pulp-2	47,545,250	45,812,386	94.34	50.55
YW-pulp-3	51,126,568	48,809,534	93.79	50.3

**Fig. 2:**
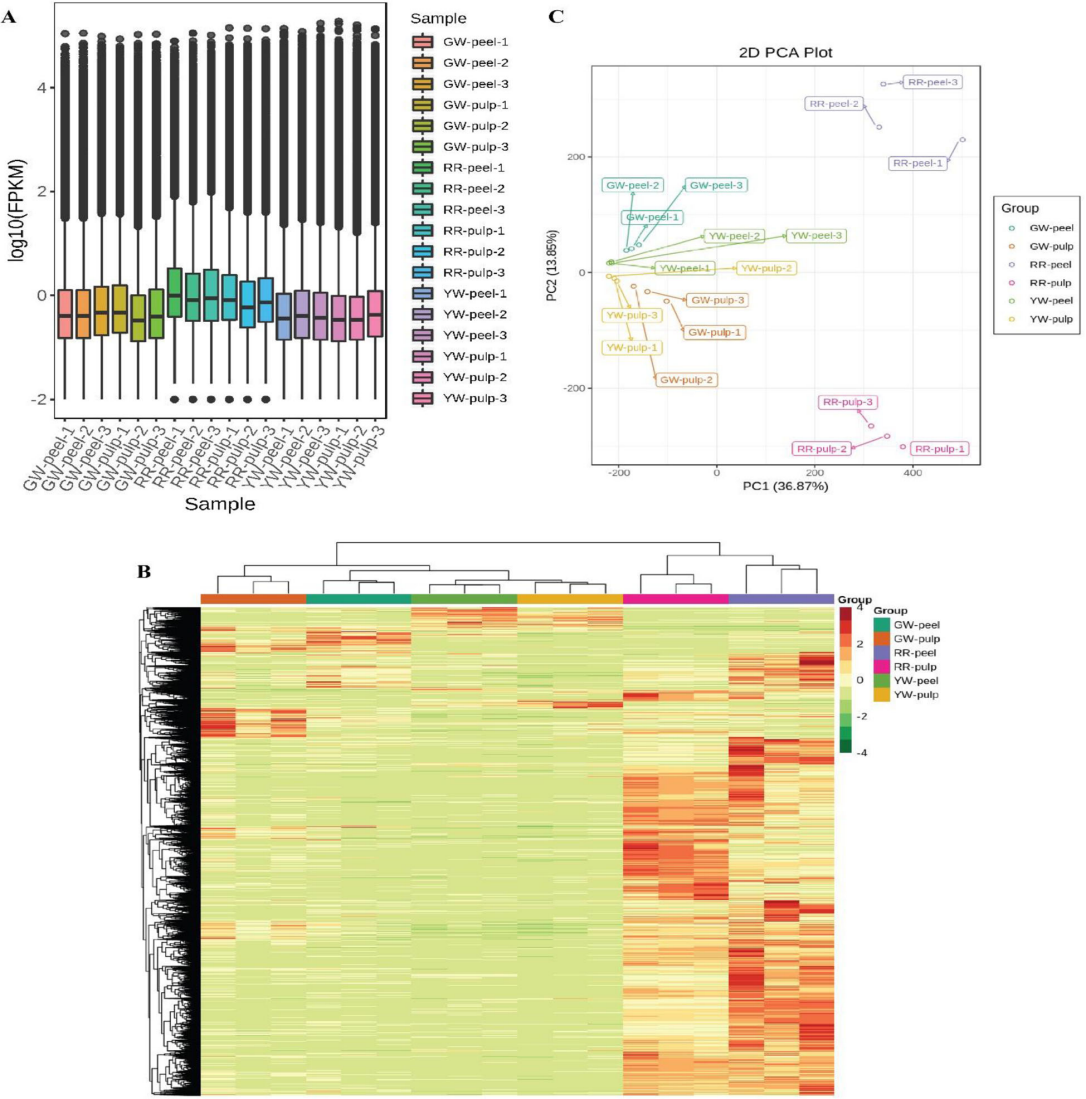
Principle component analysis of samples (**a**), and heatmap of expression (**b**). The peel and pulp colors are represented as following, Red peel and red pulp (RR), green peel and white pulp (GW), and yellow peel and white pulp (YW)

The gene expression levels of 44,263 annotated unigenes were determined in terms of fragments per kilobase of exon per million fragments mapped (FPKM) values. Principal component analysis (PCA) divided the total variation into two major components (PC1 and PC2) which contributed 36.87 and 13.85%, respectively. The PCA analysis further validated our results and showed that RR-peel and RR-pulp tissues fall away from the rest of the colored tissues (Fig. 2a). The hierarchical clustering of the samples based on FPKM values divided the six samples into two major groups. One group contains GW-peel, GW-pulp, YW-peel, and YW-pulp while the second group contains RR-peel and RR-pulp (Fig. 2b). This clustering suggests that genes encoding red color pigments mostly made a separate group from other tissues. The heatmap clearly indicated that genes in the RR-peel and RR-pulp tissues were mostly upregulated as compared to the other four tissues in which genes were mostly downregulated (Fig. 2b).

### DEGs related to betalain biosynthesis pathways in pitaya fruit

#### Differential regulation of betalain pathway in pitaya fruit peel

**Fig. 3:**
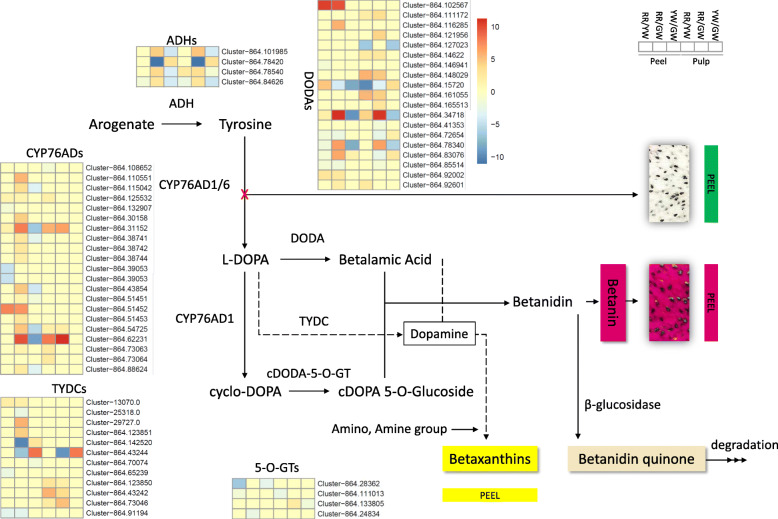
Betalain biosynthesis pathway (and tyrosine biosynthesis from arogenate). Arogenate decarboxylation catalyzed by arogenate dehydrogenase (ADH). Tyrosine 3′ hydroxylation catalyzed by cytochrome P450 enzymes (CYP76AD1/6) to form L-DOPA. The next step is the formation of betalamic acid from L-DOPA through the action of 4,5 DOPA dioxygenase (DODA), the conversion of L-DOPA to cyclo-DOPA by cytochrome P450 (CYP76AD1), glucosylation of cyclo-DOPA via the action of cyclo-DOPA 5-O glucosyltransferase, and the spontaneous condensation of betalamic acid with cDOPA 5-O-glucoside to form violet/red betacyanins. Tyrosine/DOPA decarboxylases (TYDCs) convert L-DOPA to dopamine, which leads to the formation of betaxanthins (shown as dotted lines). Peel colors are shown as respective colored boxes. Pulps are represented by cross-sections of pitaya fruit pulp pictures. The peel and pulp colors are represented as following, Red peel and red pulp (RR), green peel and white pulp (GW), and yellow peel and white pulp (YW)

The transcriptome comparison between peel colors showed the differential regulation of four ADHs in pitaya fruits. One and three ADHs were differentially regulated in RR as compared to GW and YW-peel. Three ADHs were downregulated in YW-peel when compared with GW-peel (Fig. 3; Tables S2, S3, S4). The tyrosine biosynthesis pathway is also one of the significantly enriched pathways in our results and plays a significant role in tyrosine supply for further steps of the betalain pathway [25]. We found that 41, 46, and 34 unigenes were differentially regulated between RR/GW, RR/YW, and YW/GW peels, respectively. Of these, the important ones are those encoding for aminotransferases and transaminases (EC:2.6.1.1, EC:2.6.1.5, and EC:2.6.1.9), five were tyrosine aminotransferases (EC:2.6.1.5) which directly affect the formation of tyrosine (Tables S2, S3, S4) [26, 27]. After the formation of tyrosine, cytochrome P450s (76 family, CYP760ADs) direct the synthesis of L-DOPA (L-3,4-dihydroxyphenylalanine) or cyclo-DOPA (cDOPA) [28, 29]. Therefore, we searched for CYP76ADs differentially expressed in different colored peels. Eighteen CYP76ADs were differentially expressed in the three fruit peels (Tables S2, S3, S4). Most importantly, we noticed that CYP76ADs upregulated in RR as compared to YW-peel and downregulated in YW as compared to GW-peel suggesting that for the formation of red peel color in RR fruits, these genes are upregulated. The next step is the conversion of L-3,4-dihydroxyphenylalanine (L-DOPA) to dopamine which is then either converted into miraxanthin-V (dopamine betaxanthin; yellow pigment color [30]) or into betalamic acid and finally to 2-decarboxy-betanidin [31]. We found that in RR-peel one tyrosine/DOPA decarboxylase (TYDC) was upregulated while two TYDCs were downregulated (Cluster-864.146941 and Cluster-864.91194) as compared to GW-peel (Tables S2, S3, S4). We also found the differential regulation of 3,4-dihydroxyphenylalanine 4,5-dioxygenase (DODA); five were downregulated and four were upregulated in RR-peel as compared to GW-peel. DODA cleaves the bond between carbons 4 and 5 in L-DOPA and converts it into cDOPA which is spontaneously recyclized to form betalamic acid [32]. Interestingly, a relatively larger number of DEGs related to the betalain pathway were found between RR-peel and YW-peel. Three TYDCs were upregulated and four were downregulated in RR-peel as compared to YW-peel. The YW-peel to GW-peel comparison showed the upregulation of two TYDCs and one DODA while downregulation of a TYDC and three DODAs (Tables S2, S3, S4). The main compounds that give red to violet color to fruit pulps and peels are betacyanins such as betanin, isobetanin, neobetanin, and prebetanin [33]. Hence, we searched DEGs that are involved in the final conversion of betalamic acid to betanin [34], in the peel transcriptome comparisons. A 5-O-glucosyltransferase (5-O-GT) was downregulated, while, 13 beta-glucosidases and a cDOPA 5-O-GT were upregulated in RR-peel as compared to GW-peel. The yellow color formation has been associated with betaxanthins. Betaxanthin is formed from betalamic acid by conjugating spontaneously with amino acids [34]. A search for amino acid biosynthesis-related DEGs resulted in 303, 352, and 156 unigenes in RR/GW, RR/YW, and YW/GW peel, respectively (Tables S2, S3, S4). These results suggest that the initial upregulation of ADH dictates the first step by increasing the production of tyrosine to develop red peel color. CYP76ADs allow the formation of the red peel color formation in RR as compared to GW [35] (Fig. 3).

#### Differential regulation of betalain pathway in pitaya fruit pulp

Since we had two different cultivars belonging to the same species (*H. undatus*) with a different flesh color i.e. white and red, therefore, we first compared the transcriptome of both cultivars to understand if pulp color formation is also due to the same pathway as of peel. Two ADHs were differentially regulated in red to while pulp (RR-pulp to GW-pulp); one was upregulated while the other was downregulated. Interestingly, except for one *CYPAD70* (*Cluster-864.108652*), all others were upregulated in RR-pulp as compared to GW-pulp. Three TYDCs and six DODAs were upregulated while two DODAs were downregulated. One cDOPA 5-O-GT was downregulated. Thirty beta-glucosidases were differentially expressed between the RR-pulp and GW-pulp; 16 upregulated and 14 downregulated. The comparison between RR-pulp and YW-pulp showed that three ADHs were upregulated and one was downregulated. Similar to the observation between RR and GW pulps, the RR-pulp showed the upregulation of all three CYP70ADs as compared to white pulped fruit (YW-pulp). Eight of 13 DODAs, one cDODA 5-O-GT, and 3 of 4 TYDCs were upregulated in RR-pulp as compared to YW-pulp. Finally, we compared the transcripts of the white pulps having different peel colors i.e. YW and GW. Three of the four ADHs, three of the six DODAs, and one TYDC were upregulated while a cDODA 5-O-GT was downregulated. Based on asimilar expression trend of *CYP70ADs* in red pulp (RR) as compared to the white pulp (GW and YW), we expected that *CYP70ADs* will not be regulated and found no differentially regulated genes annotated as *CYP70AD*. This observation suggests that between the red and white pulp these cytochromes play a pivotal role in the fruit pulp coloration (Fig. 3; Tables S5, S6, S7).

### DEGs related to carotenoid biosynthesis pathways in pitaya fruit

#### Differential regulation of carotenoid biosynthesis in pitaya fruit peel

**Fig. 4:**
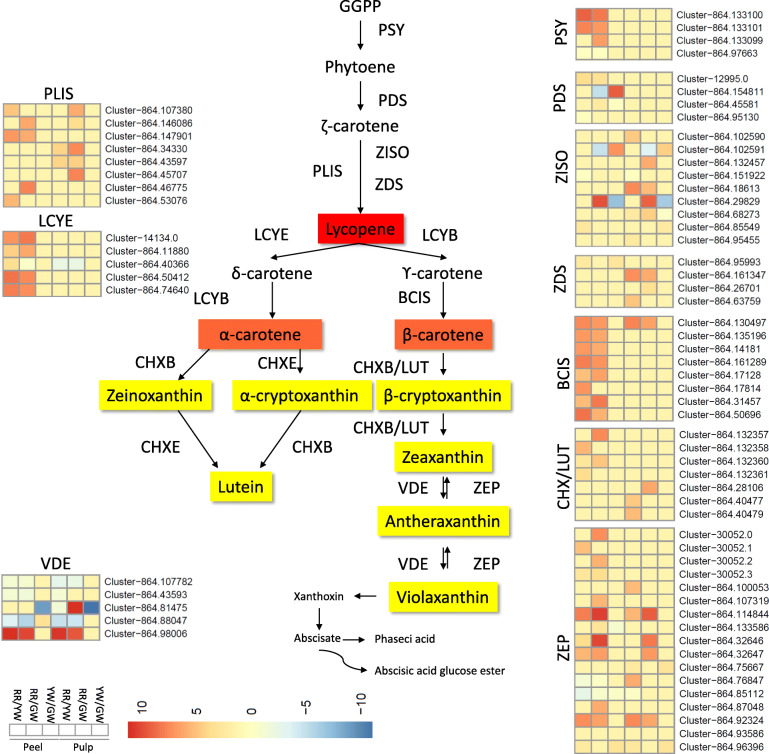
Regulation of carotenoid biosynthesis pathway in pitaya fruit pulps and peels. The pathway uses GGPP geranyl-geranyl-PP to phytoene to convert it into downstream products. The enzymes which catalyze each step in the pathways are as following, PSY phytoene synthases, PDS phytoene desaturase, ZISO ζ-carotene isomerase, ZDS ζ-carotene desaturase, PLIS prolycopene isomerases, LYCE Lycopene ξ-cyclase, LYCB lycopene β-cyclase, BCIS β-carotene isomerases, CHXB/E ζ/β -ring hydroxlases (or LUT5), ZEP zeaxanthin epoxidase, and VDE violaxanthin de-epxidase. The peel and pulp colors are represented as following, Red peel and red pulp (RR), green peel and white pulp (GW), and yellow peel and white pulp (YW)

Two and three phytoene synthases (PSY) were upregulated in RR-peel as compared to GW-peel and YW-peel, respectively, while one was downregulated in the same. No differential expression of PSYs was detected in YW to GW-peel. PSY converts geranyl-geranyl-PP to phytoene which is then converted into phytofluene and ξ-carotene by phytoene desaturase (PDS) [36]. One PDS was upregulated while another was downregulated in RR-peel as compared to GW-peel, however, two were upregulated and one was downregulated in RR-peel as compared to YW-peel. We also found two differentially regulated PDSs in YW to GW peel; one up- and one downregulated. The upregulated PDS (*Cluster-864.154811*) had expression close to zero in GW. One ζ-carotene isomerase (ZISO) was upregulated and one was downregulated in RR-peel as compared to GW-peel, while, the same was upregulated in the case of RR-peel and YW-peel, however, the genes encoding ZISOs were different. The two ZISOs that were differentially regulated between RR and YW-peel, were differentially regulated between YW and GW peel but their expression was the opposite. The ZISO converts 9,15,9′-tri-cis-ζ-carotene to 9,9′-di-cis-ζ-carotene and then ζ-carotene desaturase (ZDS) converts it to 7,9,9′-tri-cis-neurosporene and then to 7,9,9′-tetra-cis-neurosporene [37]. One ζ-carotene desaturase (ZDS) was upregulated between RR and GW peel, and YW and GW peel. Three prolycopene isomerases (PLIS) were upregulated between each RR and GW peel, and RR and YW peel, however, the genes encoding PLIS in each comparison were different. It is important to consider that the expression of these genes was nearly zero, hence, it may be suggested that these genes did only express in RR peel. PLIS converts the product of ZDS to lycopene. Lycopene ξ-cyclase (LCYE), which converts lycopene to carotene was upregulated in RR as compared to GW as well as YW peel and there was no differential regulation of LYCEs between YW and GW peels [36]. Eight and seven β-carotene isomerases (BCIS) were upregulated in RR peel as compared to GW and YW peel, respectively. No BCIS was differentially regulated between YW and GW peel. We found beta-ring hydroxylases (CHX) that were mapped on the KEGG pathway as LUT5 and were converting β-carotene to β-cryptoxanthin and zeaxanthin [36, 37]. These genes were upregulated in RR peel as compared to GW and YW peels. The genes which are involved in the reversible conversion of zeaxanthin to antheraxanthin and then to violaxanthin; zeaxanthin epoxidase (ZEP) and violaxanthin de-epoxidase (VDE) [38], were also differentially regulated between RR and GW, and RR and YW peels. Most of ZEPs were upregulated in RR peel as compared to GW and YW peels except for two that were downregulated. VDEs were upregulated in RR peel as compared to GW and YW peel. One VED (*Cluster-864.131514*) was downregulated in RR as well as YW peel as compared to GW peel. Two of three ZEPs were downregulated in YW peel as compared to GW peel. Interestingly, genes that are involved in the formation of xanthoxin, abscicate, and abscisic acid glucose ester were also differentially regulated between pitaya peels (Fig. 4).

#### Differential regulation of carotenoid biosynthesis in pitaya fruit pulp

No PSY or PDS was differentially expressed in pitaya fruit pulp. Four ZISOs were upregulated in RR-pulp as compared to each GW and YW-pulp. Two ZISOs were upregulated while two were downregulated YW-pulp as compared to GW-pulp. Two ZDSs were upregulated in RR-pulp as compared to GW and YW-pulp, while no ZDS was differentially regulated between YW and GW-pulp. The expression of PLISs was similar to that of peel i.e. upregulation in RR-pulp as compared to GW and YW-pulp but no differential expression between YW and GW pulp. One LCYE was downregulated and one BCIS was upregulated in RR as compared to GW and YW-pulp. ZEPs and VDEs were upregulated in RR-pulp as compared to GW and YW-pulp. Two ZEPs were also upregulated in YW-pulp as compared to GW-pulp. The genes related to the formation of xanthoxin were downregulated in RR-pulp as compared to GW and YW-pulp with the exception of the upregulation of two genes. Similar to peel, we also noticed the differential expression of genes in the pulp, which convert xanthoxin to abscicate and abscisic acid glucose ester. However, most of the genes were regulated in RR-pulp in comparison to GW and YW-pulp and a limited category of genes were regulated between YW and GW-pulp (Tables S5, S6, S7).

Overall, the transcripts related to the carotenoid pathway showed no or very limited differential regulation between YW and GW-pulp, and some genes which control the important steps in the pathway i.e. PYS, PDS, LCYE, and BCIS were absent in pulp with the exception of one LCYE and BCIS. Most of the genes in the carotenoid pathway were not differentially regulated between YW and GW pulp indicating limited or no role of carotenes in pitaya fruit peel yellow coloration (Fig. 4).

### DEGs related to anthocyanin biosynthesis pathways in pitaya fruit

#### Differential regulation of anthocyanin biosynthesis in pitaya fruit peel

Our results demonstrated that anthocyanin biosynthesis was one of the significantly enriched pathways. Hence, we searched for DEGs implicated in important steps in this pathway. A phenylalanine ammonia-lyase (PAL) gene (*Cluster-864-82,920*) was found in our transcriptome, however, in peel, it wasn’t differentially regulated. One of the two trans-cinnamate 4-monooxygenases (C4H) was upregulated in RR-peel as compared to YW-peel while downregulated in YW as compared to GW-peel. The expression of the C4H gene in YW-peel was almost zero in both comparisons (0.23 FPKM). We observed that in RR-peel, as compared to GW-peel, four 4CLs (4-coumarate-CoA ligases) were downregulated while three of the four 4CLs were upregulated in RR-peel as compared to YW-peel. Two DEGs annotated as 4CHs were downregulated in YW-peel as compared to GW-peel. Most of the chalcone synthases (CHSs) were downregulated in RR-peel as compared to GW and YW peel except two genes (*Cluster-864.109275* and *Cluster-864.98602*) that were actually upregulated in RR-peel as compared to other two peel colors. None of the detected CHSs was differentially regulated between YW and GW-peels. We found 10 differentially regulated chalcone isomerases (CHIs) in our transcriptome datasets. Five and three CHIs were downregulated in in RR-peel as compared to GW and YW-peels, respectively. Two of the CHIs were upregulated in RR-peel as compared to GW-peel. Two of the three were downregulated in YW-peel while one was upregulated as compared to GW-peel. The anthocyanidin 3-O-glucoside 2′-O-xylosyltransferase (UFGT) which contributes to the last few steps of the anthocyanin biosynthesis pathway were also differentially regulated in our transcriptome datasets [39]. One UFGT was downregulated while two were upregulated in RR-peel as compared to GW and YW-peels, respectively. One UFGT was downregulated in YW-peel as compared to GW-peel. Other genes such as flavonol synthase (FLS), bifunctional dihydroflavonol 4-reductase (DFR), leucoanthocyanine reductase (LANR), and anthocyanin reductase (ANR) were also differentially regulated between different peel types (Tables S2, S3, S4).

#### Differential regulation of anthocyanin biosynthesis in pitaya fruit pulp

**Fig. 5:**
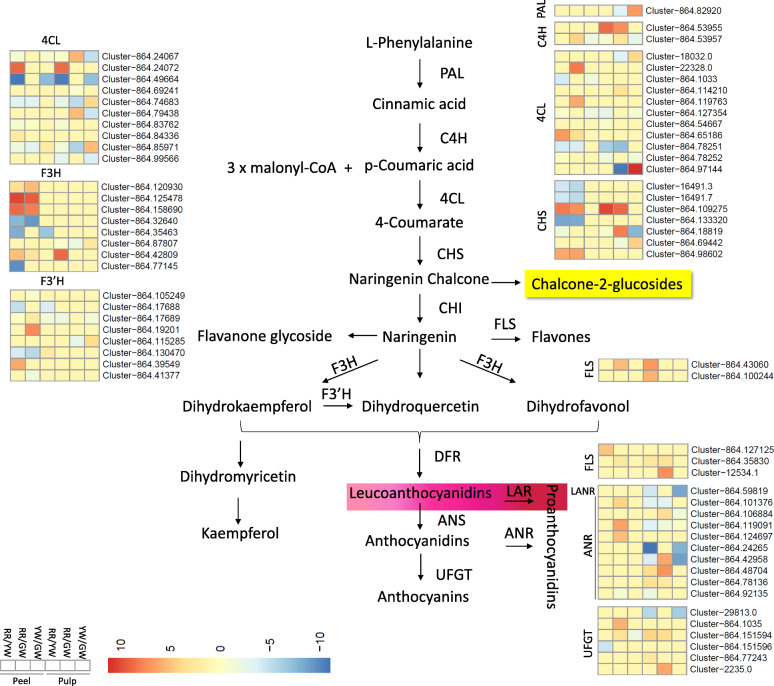
Regulation of anthocyanin biosynthesis pathway in pitaya fruit pulps and peels. PAL phenyl ammonia-lyase, C4H cinnamate 4-hydroxylase, 4CL 4-coumarate-CoA ligase, CHS chalcone synthase, CHI chalcone isomerase, F3H flavanone 3-hydroxylase, DFR dihyroflavonol 4-reductase, ANS anthocyanin synthase, UFGT UDPG-flavonoid-3-O-glucosyltransferase, LAR leucoanthocyanidin reductase, FLS flavonol synthase, ANR anthocyanidin reductase, and LANR leucoanthocyanidin reductase. The peel and pulp colors are represented as following, Red peel and red pulp (RR), green peel and white pulp (GW), and yellow peel and white pulp (YW)

The pulp transcriptome comparison showed that one PAL was downregulated in RR as compared to YW-pulp and upregulated in YW as compared to GW-pulp. One C4H was upregulated in RR as compared to GW and YW-pulp while it was not differentially expressed between YW and GW-pulp. The second gene was downregulated in RR and YW-pulp as compared to the GW-pulp and upregulated in RR as compared to YW-pulp. Four, three, and two 4CLs were downregulated in RR to GW, RR to YW, and YW to GW-pulp, respectively. However, we also noticed the upregulation of one and two 4CHs in RR to YW, and YW to G-pulp, respectively. Among CHSs, one gene was upregulated in RR as compared to GW as well as YW-pulps, while another gene was downregulated in RR to GW and YW to GW-pulp and upregulated in RR to YW pulp. All CHIs, except one, were downregulated in RR as compared to GW-pulp. Three of five were downregulated in RR as compared to YW pulp and four of six were downregulated in YW as compared to GW-pulp. UFGTs were upregulated in RR as compared to GW and YW-pulp, while downregulated in YW to GW pulp (Fig. 5; Tables S5, S6, S7).

### Transcription factors

The pitaya fruit peel and pulp showed the differential regulation of TFs belonging to 80 different families. The major number of the differentially expressed TFs belonged to AP2/ERF, AUX/IAA, bHLH, bZIP, C2H2, C3H, and MYB families (Table S8). MYB TFs are major components in the provision of definite gene expression patterns and are associated with biosynthetic pathways of secondary metabolites and flavonoid metabolism [40, 41]. Particularly, the R2R3 subfamily in fruits has been associated with flavonoid biosynthesis [42, 43]. Furthermore, bHLH proteins and WD40 repeat-containing genes together with the MYB TFs form anthocyanin biosynthesis regulatory complex [44]. Hence, we specifically searched these TFs and genes in our comparative transcriptome datasets. We found differential regulation of 72, 34, and 37 transcripts annotated as MYB TFs, bHLH TFs, and WD40 repeat-containing genes. Of the MYB TFs, we specially searched for R2R3 subfamily members and found three transcripts (*Cluster-10,155*.0, *Cluster-864.101811*, and *Cluster-864.67362*). The first of the three i.e. *Cluster-10,155.0* was annotated as R2R3-MYB flavonol regulator according to Tremble. Its expression in RR-peel was higher (FPKM = 2.50) while its expression was almost zero i.e. 0.1 and 0.05 in GW and YW-peel, respectively. However, its expression was not detected in pulps. The other two had higher expressions in pulps as compared to respective peels, with the maximum in RR followed by GW and YW-pulp. Thirteen bHLH137-like TFs were expressed exclusively in RR as compared to YW-peel while one (*Cluster-864.167690*) was not expressed at all in both YW and GW-peels. Same as peel, twelve bHLH TFs were exclusively expressed in RR as compared to YW pulp and four of these also did not express in GW-pulp, suggesting that these four TFs are RR specific and are important candidate genes for future studies. Three WD40 domain-containing proteins were RR-peel specific while none was YW-peel specific. We also noticed WD40 domain-containing proteins, which had no or very low expression in GW and YW-pulps.

### Validation of key DEGs involved in peel and pulp color by qRT-PCR

**Fig. 6:**
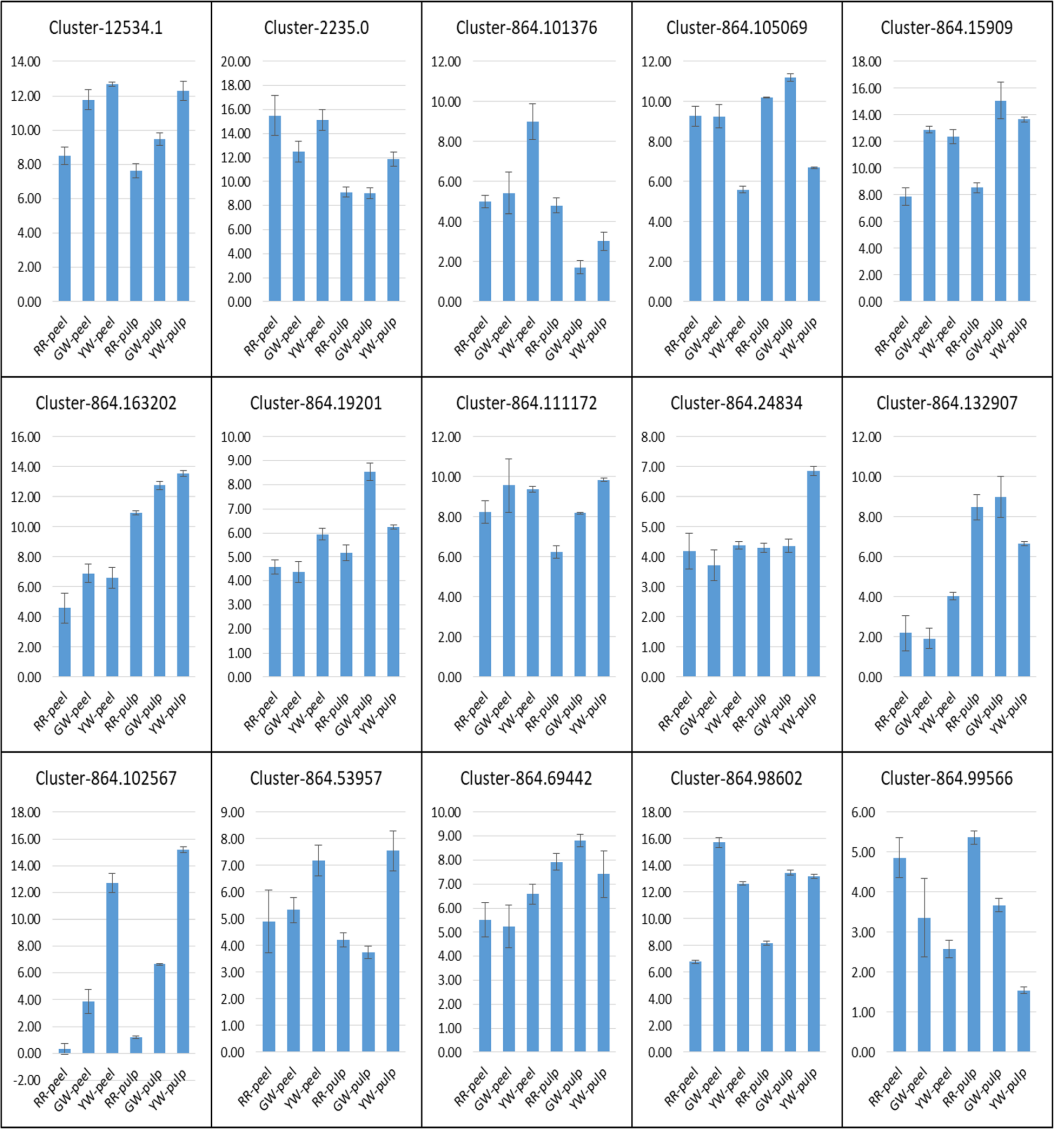
qRT-PCR validation of selected unigenes. The x-axis represents the tissue type while the y-axis represents the relative expression of each gene. The bars show standard deviation. The peel and pulp colors are represented as following, Red peel and red pulp (RR), green peel and white pulp (GW), and yellow peel and white pulp (YW)

To validate the relative expression pattern of the unigenes, we selected 15 key DEGs related to anthocyanin and betalain biosynthesis. We confirmed the expression pattern of these genes using qRT-PCR assay, which has validated our RNA-seq data and the selected genes showed similar expression profiles as observed in transcriptome data (Fig. 6).

### Differentially accumulated metabolites in pitaya fruit pulp and peel

**Fig. 7:**
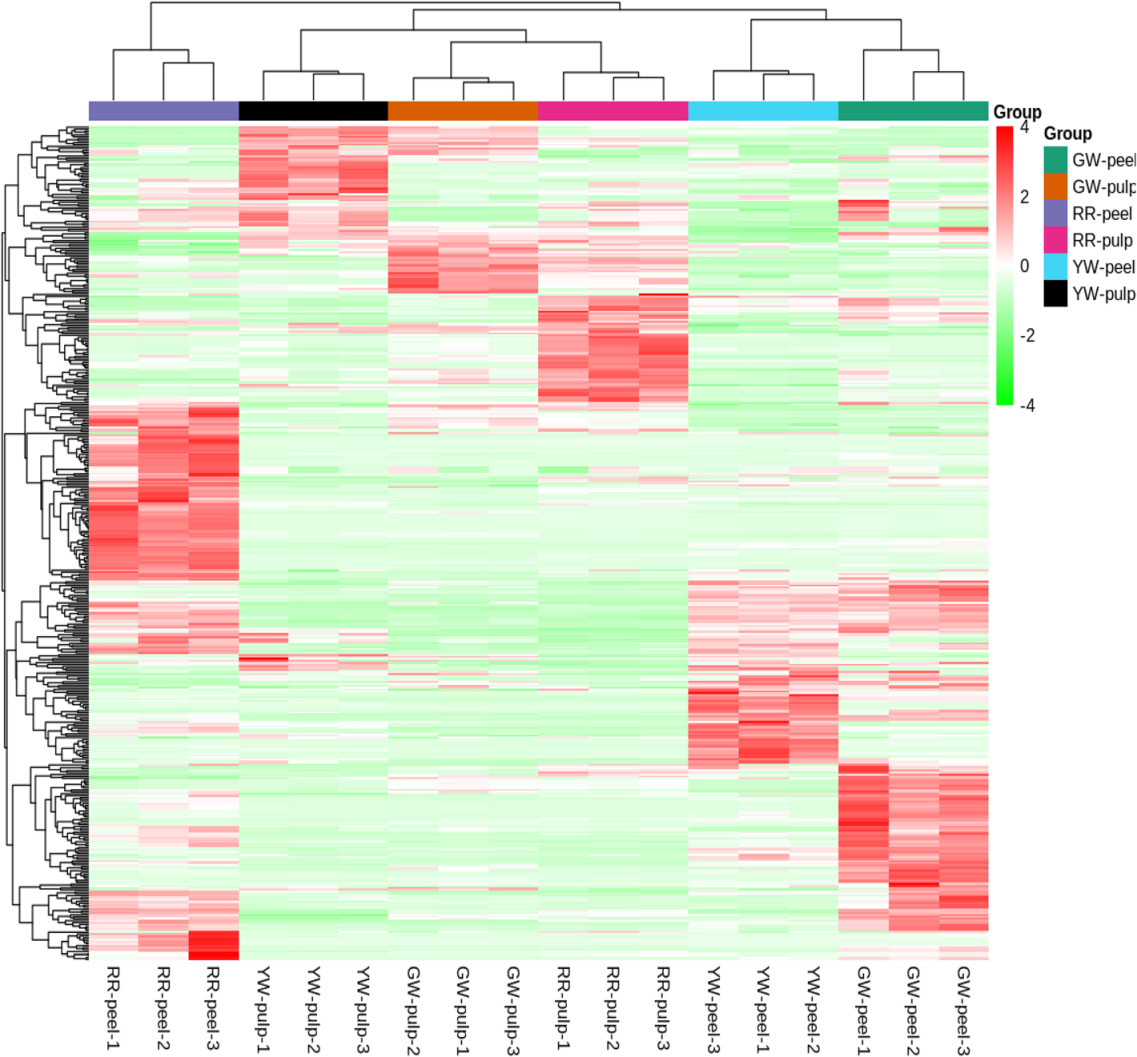
Heatmap of differentially accumulated metabolites in different colored pitaya fruit peels and pulps. The peel and pulp colors are as following, Red peel and red pulp (RR), green peel and white pulp (GW), and yellow peel and white pulp (YW)

The general metabolite profiles of the studied pitaya fruits with different colored peels and pulps showed marked differences. A total of 232, 242, 217, 159, 202, and 175 were differentially accumulated between RR and GW-peel, RR and YW-peel, YW and GW-Peel, RR and GW-pulp, RR and YW-pulp, YW and GW-pulp, respectively (Fig. 7).

Twelve different alkaloids were differentially accumulated in peels. Of these, important ones were the betalains. Gomphrenin-I was accumulated in RR and GW-peel, while no accumulation in YW-peel. Similar to peels, it also showed accumulation in RR and GW pulp with a very limited quantity in GW-pulp, while a very high concentration in RR-pulp. Phyllocactin-II had a similar pattern where it showed high accumulation in RR peel and pulp. The RR-pulp had 7.6 fold higher phyllocactin-II content than RR-peel. It had a very low concentration in the YW-peel (− 908 fold) as compared to RR-peel and was not detected in YW-pulp. The concentration in GW-pulp was also close to YW-pulp i.e. -7166 fold as compared to RR-pulp. Isophyllocactin was also accumulated in high quantities in RR-peel as compared to YW and GW-peel. YW and GW-pulp had no or very low isophyllocactin concentrations, respectively. Amaranthine was accumulated in higher quantities only in RR-peel as compared to GW and YW-peels which showed no or very low accumulation, respectively. It did not follow the same trend as of the above-mentioned metabolites in peel and pulp as we did not detect higher quantities in RR-pulp. Betanin which has been previously reported to be an important component of pitaya red pitaya fruit peel and pulp [2, 5] was present in low quantities in RR-peel as compared to GW and YW-peels. It was not detected in YW-pulp and the quantity in GW-pulp was almost double than RR-pulp. This is possibly due to the conversion of betanin into gomphorenin-I as the latter was the significantly enriched metabolite mapped on the betalain biosynthesis pathway [45]. In the upstream, we also detected that L-tyrosine metabolism-related metabolites were also differentially accumulated. Most importantly, we detected the upaccumulation of *p*-coumaric acid in RR as compared to GW and YW-peels, YW as compared to GW-peel, and YW as compared to GW-pulp. It was downaccumulated in RR as compared to GW-pulp. L-tyrosine, was upaccumulated in RR as compared to GW-peel, YW as compared to GW-peel, RR as compared to YW-pulp, while it was downaccumulated in RR as compared to GW-pulp.

As transcriptome analysis showed differential regulation of genes associated with the anthocyanin pathway, we also detected the accumulation of anthocyanins in pitaya pulp and peel. Overall, 12 and 70 different anthocyanins were differentially accumulated in peels and pulps, respectively. Cyanidin 3-O-galactoside, Cyanidin 3-O-malonylhexoside, Cyanidin O-syringic acid, Malvidin 3-O-galactoside, and Petunidin 3-O-glucoside were peel specific metabolites while the other seven were both in peels and pulps. Cyanidin 3-O-galactoside was 22.6 and 27.3 fold up-accumulated in RR as compared to both GW and YW-peels, respectively. It was only found in RR-pulp and was absent in GW and YW-pulps. Cyanidin 3-O-glucoside (Kuromanin) showed upaccumulation in RR-peel as compared to GW and YW peels, and in RR-pulp as compared to GW-pulp. In white pulps, its quantity was very low (YW) or zero (GW). Cyanidin 3-O-malonylhexoside was downaccumulated in RR as compared to GW and YW-peels, and in YW as compared to GW-peel. Cyanidin 3-rutinoside (Keracyanin chloride) was ~ 71 fold higher in RR-peel as compared to YW peel while GW-peel didn’t contain this metabolite. Interestingly, it had a 2.64-fold higher concentration in RR as compared to GW-pulp and wasn’t present in YW-pulp. Cyanidin chloride was downaccumulated in RR as compared to GW-peel and in YW as compared to GW peel. It also showed downaccumulation in RR pulp as compared to GW and YW-pulp while no differential accumulation between YW and GW-pulps. Cyanidin -O-glucoside-O-rhamnoside had 47 and 295 fold higher accumulation in RR as compared to GW and YW-peel. This metabolite was not absent in YW-pulp while its concentration was three-fold higher in RR as compared to GW-pulp. Malvidin 3-O-galactoside was not differentially accumulated between RR and GW-peels, while it was upaccumulated in RR as compared to YW-peel. It was down accumulated in YW as compared to GW-peel. A similar trend was observed for oenin chloride. Petunidin-3-O-glucoside was only upaccumulated in RR peel as compared to GW peel. Petunidin was absent in GW-peel and RR-pulp while its concentration in white pulps and yellow peel was quite low as compared to red-peel. We searched for YW-peel specific metabolites and found two flavonoids (cyanidin O-syringic acid and 6-C-Hexosyl-hesperetin O-hexoside), one organic acid (citric acid), and two phenolic acids (isochlorogenic acid A and verbascoside). The most important observation was 213 and ~ 30 fold higher concentration of luteolin-7,3′-Di-O-β-D-Glucoside in YW-peel as compared to RR and GW-peels, respectively (Table S9).

Together, these metabolic responses suggest that the major components of red peel are Gomphrenin I, Phyllocactin II, isophyllocactin, amaranthine, Cyanidin -O-glucoside-O-rhamnoside, Cyanidin 3-rutinoside (Keracyanin chloride), Cyanidin 3-O-galactoside, Cyanidin 3-O-glucoside (Kuromanin), and Petunidin. The YW and GW-peels’ anthocyanin comparison suggested that Cyanidin O-syringic acid is accumulated in YW-peel, which is an anthocyanin degradation product. Betanin, malvidin 3-o-galactoside, and oenin chloride are degraded in YW-peel as compared to RR and GW-peels.

## Discussion

Peel and pulp color is an important trait for pitaya fruit due to its consumer attraction and nutritional value. Although studies were conducted to elucidate its molecular and metabolic mechanism using either transcriptome or metabolome data, a combined approach has not been used. Integrating transcriptome and metabolome data can better explain the key metabolites involved in pitaya color formation and its molecular basis. In this study, we performed a combined transcriptome and metabolome analysis to identify key genes and metabolites associated with peel and pulp color formation in three pitaya species. Recently, it was reported that the color-breaking stage in pitaya fruit is 26 DPA [3]. Considering this color-breaking stage, we sampled the fruits of three pitaya cultivars at 30 DPA; two of which belong to the same species while the third belongs to another species.

### Pulp color formation in pitaya fruit

The colors of the pitaya fruit pulp are conferred by different types of water-soluble pigments [3, 5]. Recently, a number of studies have used different omics approaches to identify the key regulatory genes and pathways in pulp color formation. One common result in those studies is the regulation of the betalain biosynthesis pathway in pulp and peel. For example, Hua et al., [46] identified CYP76ADs and DOPA proteins through proteomic analysis of the pitaya pulp and that the betalain pathway was significantly enriched between white and red pulps. Another study by the same group on metabolomic characterization of different pulp colors revealed the same results [2]. However, the color-breaking stage between both pulp colors has not been discussed concerning the key discriminant step in the pathway. It has been reported that betalains, but not the anthocyanins, are responsible for the red pulp color of [5]. Our results that ADH expression in red pulp was increased as compared to white pulp suggest that the first strategy to develop red pulp is to increase the starting material of the pathway i.e. tyrosine, which was also evident from metabolome analysis [25]. Furthermore, the upregulation of all CYP76ADs in red pulp as compared to white pulp regardless of white pulp containing pitaya species suggests that it is the color-breaking stage which decides whether the pulp will gain red color or not. It is previously known that the expression of *CYP76ADs* in red pitaya pulp increases to many folds at 30 DAP as compared to the initial transcript level before color-breaking in *H. polyrhizus* [35]. Therefore, based on this observation of *CYP76ADs* expression, and the results observed by metabolome analysis that major betanin pathway products were accumulated in the red pulp clearly indicate that this step determines whether the pulp will remain colorless (white) or gain color. In order to further understand the regulation of *CYP76ADs*, we searched for *WRKY44s* that were expressed in our transcriptome datasets between different pulp colors because it is known that in *H. polyrhizus* the *CYP76s* are regulated by *WRKY44s* [35]. We found *WRKYTF44* (*cluster-864.33879*) which showed higher expression in RR-pulp (17.05 FPKM) as compared to GW and YW-pulps (6.2 and 0 FPKM, respectively) (Fig. 3). Apart from betalains, it has been previously known that betalains occur in a mutually exclusive fashion with anthocyanins, as no plant has been found to naturally produce both types of pigments [47]. However, a previous transcriptome based study detected differential regulation of the anthocyanin biosynthesis-related genes [5] but the authors justified that the anthocyanin biosynthesis pathway related genes (homologues) were shorter than the reference sequences. Another study targeting *H. polyrhizus* proteome reported the enrichment of two anthocyanin biosynthesis related proteins in their results along with betalain biosynthesis related proteins [46]. Therefore, based on these reports, the presence of anthocyanins can be speculated. The comparative pitaya transcriptome showed the differential regulation of the anthocyanin pathway and genes controlling almost every single step in the pathway were differentially regulated (Fig. 5; Table S2, S3, S4, S5, S6 and S7). In accordance with the transcriptome datasets, the metabolome analysis clearly showed the accumulation of anthocyanins in red pulps as compared to the white pulp (Table S9). We ranked the metabolites of both pathways in descending order and found that in the top-6 metabolites, two were the anthocyanins. Therefore, these observations are not ignorable [48]. One study by Nakatsuka et al., [49] reported that non-betalain-producing plant and fungal species can be engineered for betalain production. These observations need further targeted studies for a better understanding of the co-pigmentation of these compounds in pitaya fruit. An early understanding could be generated by computational models based on the dispersion-corrected density functional theory [50].

In any case, no color-breaking stage for the development of anthocyanins in red and no biosynthesis of anthocyanins in white pulp were found. Together, we propose that red pitaya pulp color is under the strict regulation of *CYP76ADs* by WRKYs and the anthocyanin coexistence with betalains is unneglectable (Fig. 3).

### Peel color formation in pitaya fruit

Studies on pitaya fruit peel coloration have reported that degradation of chlorophyll and betalain biosynthesis is what turns peels red and the betanins’ content starts significantly increasing from 26 DAP [3]. Therefore, in order to stay green, the fruit peel must not start excessive betanins’ biosynthesis. The upregulation of *CYP76ADs* in red peels as compared to green and yellow peels suggests that regulation of red peel color is quite similar to the pulp. Very low but smaller expression of *CYP76ADs* in green peel is in accordance with the observation that betalains are present in pitaya fruit peels even before the color-breaking stage [3]. Similar to transcripts, we also noticed minor quantities of betalains in metabolome suggesting that pitaya fruit peels contain betalains in minor quantities in green peels as well but the color-breaking stage increases their concentrations in red peels. All observed *CYP76ADs* were downregulated in yellow peel as compared to red as well as green peels. These observations suggest that betalain biosynthesis gives red coloration to pitaya peels and their no significant accumulation even after the color-breaking time keeps the skin green. In case of yellow skin color, no particular studies explained the yellow peel color formation in pitayas and most of the research reported that it is due to yellow betalain pigments [11]. To explore the key genes involved in yellow peel color formation, we additionally explored anthocyanin as well as carotenoid biosynthesis pathways owing to earlier reports of their involvement in yellow peel formation in different fruits [51, 52]. We first confirmed if yellow coloration related metabolites were differentially detected or not. Firstly, the *CYP76ADs* were downregulated in yellow peel as compared to both green and red peels suggesting that the tyrosine conversion to L-DOPA in yellow peel is not being regulated at the same extent as of other peel colors. The metabolome also showed same results, where dopamine hydrochloride concentration was highest in red, followed by green, and yellow. This means that dopamine is being formed in all peel types, which is evident from the differential regulation of TYDCs in all peels (Fig. 3). The final product for yellow coloration should be either 3-methoxytyramine-betaxanthin or miraxanthin-V [53, 54]. The former is produced by the action of catechol o-methyltransferase (EC:2.1.1.6) [53] but we did not find any gene encoding this enzyme in the DEGs data. The latter is produced by spontaneous conversion of dopamine by interacting with betalamic acid (dopa-xanthin) [28]. The lower concentration of dopamine in yellow peel also dictates that possibly it has been used for the formation of betaxanthins. We propose this because of two reasons. First, two of the three TYDCs were upregulated in yellow peel as compared to green and red peels which lead us think that yellow color formation is possibly due to betaxanthin formation. Second, this could be further supported by the fact that a putative 2-aminoindan 2-phosphonic acid gene (AIP, *Cluster-864.78979*) was significantly upregulated in yellow peel as compared to green peel but was not differentially regulated between red and yellow peel as well as red and green peel. AIP is a strong inhibitor of PAL and has been experimentally used to increase endogenous (*S*)-Phe levels and subsequently the formation of (*S*)-Phe-betaxanthin in golden beet, red beets, broad bean, and pea [28].

We further explored the possibility of yellow color forming anthocyanin presence in the peels’ metabolites. The only possibility is the production of chalcone-2-glucosides, which are synthesized from narigenin chalcone [55]; which is itself gives yellow color to tissues. Firstly, the CHS genes were not differentially regulated between yellow and green peels, which means that they could not be involved higher naringenin chalcone production and its conversion into chalcone-2-glucosides [56, 57]. Secondly, in the yellow peels’ metabolome, we did not detect naringenin chalcone. Therefore, based on these results, the possibility of involvement of anthocyanins in yellow color formation could be ruled out (Fig. 5).

Finally, we sought to see if carotenoids are involved in yellow peel coloration as reported in different flowers and fruits [36, 37]. In this regard, our results that one PDS, four ZISOs, and one ZDS differentially expressed suggest that carotene biosynthesis pathway is being regulated [37], however no LCYE or CHXs were differentially expressed between yellow and green peel. This clearly suggests that carotenoid pathway might not be involved in yellow peel color formation. It was further supported by the fact that carotenoid pathway was not significantly enriched between metabolite comparisons (Fig. 4). Therefore, our results are in agreement with previous reports that betalains are the main pigments for the peel and pulp color formation in pitaya fruit [2, 3, 5, 46].

## Conclusion

In summary, we applied a combination of transcriptome sequencing and metabolome profiling approach to understand the possible involvement of different pigments biosynthesis pathways in pitaya fruit color formation. We found that red and yellow pitaya peel color formation is mainly due to betalain biosynthesis pathway. We propose that the key breaking stage for the color formation from green peel could be the activation of the betalain biosynthesis pathway where the expression of *CYP76ADs* is enhanced by WRKY TFs. The yellow peel contained both betalain pathway and anthocyanin biosynthesis pathway related metabolites, suggesting a relatively complex mechanism and needs further exploration. The pulp color formation is also due to betalain biosynthesis. Thus, the metabolism of major pathways and their regulatory genes was elucidated in this comparative study, which provides valuable genomic resources for pitaya fruit color breeding. Although we have identified many candidate genes for color formation using transcriptome analysis, further studies for the functional characterization of these candidate genes are required which may employ CRISPR/Cas9 for rapid and targeted genome editing [58].

## Methods

### Plant materials, cDNA preparation and Illumina sequencing

Three different types of pitaya fruits were collected from “Sanya fruit Island, Dragon fruit planting base, Sanya, Hainan, China” on June 20, 2019. The fruits include: (i) yellow-skinned and white-pulp (YW) pitaya (*Hylocereus megalanthus*), (ii) Red-skinned and red-flesh (RW) pitaya (*Hylocereus undatus*), and (iii) Green-skinned and white-fleshed (GW) pitaya (*Hylocereus undatus*) (Fig. 1). The formal identification of the plant materials was undertaken by the corresponding author of this article (Professor Rulin Zhan). No voucher specimen of this material has been deposited in a publicly available herbarium. The total RNA was extracted from peel and pulp tissues of three types of pitaya fruits with three independent biological replicates using the Tiangen RNAprep Pure Plant Kit (Tiangen, China). The quality of the total RNA was checked by agarose gel electrophoresis and the concentration of the total RNA was determined by NanoDrop (Thermo Scientific, USA). The 18 cDNA libraries were prepared using NEB Next Ultra RNA Library Prep Kit following manufacturer’s instructions. The mRNA was purified from total RNA of each of three replicate using poly-T oligo-attached magnetic beads and then broken into short fragments to synthesize first-strand cDNA. The second strand cDNA synthesis was subsequently performed using DNA Polymerase I and RNase H. After end repair, adaptor ligation, and the addition of index codes for each sample, PCR amplification was conducted using High Fidelity DNA polymerase with universal PCR primers. The cDNA library products were sequenced by Illumina paired-end sequencing technology with read lengths of 100 bp, and they were sequenced on an Illumina HiSeq 2000 instrument by Wuhan Metware Biotechnology Co., Ltd., Wuhan, China.

### Sequencing, data filtering, and De novo assembly

Before performing data analysis, the raw reads were subjected to quality check by FastQC (http://www.bioinformatics.babraham.ac.uk/projects/fastqc/). The low-quality reads were removed using Trimmomatic version 0.33 from raw paired-end reads to obtain high-quality clean reads. Low-quality sequences include reads with adapters or those with N content exceeding 10% of the number of read bases. After purity filtering was completed, the high-quality reads were assembled by Trinity with default parameters to construct unique consensus sequences [59].

### Gene expression and analysis of differential expressed genes

The Unigene expression levels were calculated in terms of fragments per kb per million reads (FPKM) values. The differential gene expression analysis was performed by R package DESeq2 [60, 61] using unstandardized reads count data as input. The false discovery rate (FDR) method was introduced to determine the threshold *p*-value at FDR ≤ 0.05; the absolute value of |log2Ratio| ≥ 1 was used as the threshold to determine the significance of the differential expression of Unigenes.

### Gene annotation and classification

To perform functional annotation, the assembled Unigenes were submitted to a public database and compared with the NCBI non-redundant protein database (Nr), NCBI nucleotide sequence database (Nt), Swiss-Prot (http://www.uniprot.org/) [62], Kyoto Encyclopedia of Genes and Genomes (KEGG) databases (http://www.genome.jp/kegg/) [63], and KOG (ftp://ftp.ncbi.nih.gov/pub/COG/KOG) [64] using blastx (v.2.2.26) [65].

The gene ontology (GO) annotations were analyzed using the Blast2GO (V.2.5) program (http://www.geneontology.org) [66]. All differentially abundant Unigenes between different samples were mapped to the GO and KEGG pathway databases, and then the respective number of Unigenes for each GO and KEGG orthology (KO) terms were calculated. To compare these Unigenes with the whole transcriptome background from pitayas, significantly enriched GO and KO terms from the set of differentially abundant Unigenes were identified using the hypergeometric test [67].

### Metabolic profiling

The biological samples consisting of peel and pulp were placed in a lyophilizer (Scientz-100F) under vacuum freeze-drying and ground to a fine powder. Samples of about 100 mg powder were weighed and dissolved in 0.6 mL of 70% methanol. Samples were kept in a refrigerator at 4 °C overnight, and vortex six times during the period to increase the extraction rate. After centrifugation (rpm 10,000 g, 10 min), the supernatant was filtered through a microporous filter (0.22 μm pore size), proceeded for Ultra Performance Liquid Chromatography (UPLC)- mass spectrometry (MS / MS).

For secondary metabolic profiling, about 0.1 g freeze-dried powder of peel or pulp was extracted with 80% methanol. Secondary metabolic profiling was performed using a QTOF 6520 mass spectrometer (Agilent Technologies, Palo Alto, CA, USA) coupled with a 1200 series Rapid Resolution HPLC system. Secondary metabolites and other amino acids were identified by comparing the characteristic fragment ion with a reported reference [1, 68–70] and metabolites with similar fragment ions were suggested to be the same compounds.

### Statistical analysis

Statistical analyses were conducted by uploading the secondary metabolites data on the Analyst 1.6.1 software (AB SCIEX, Ontario, Canada). Partial least squares-discriminant analysis was applied to calculate the corresponding variable importance in projection (VIP) value. When the VIP ≥ 1, and fold change ≥2 or fold change ≤0.5, the metabolites were considered as differentially changed metabolites. Principal Component Analysis (PCA) was conducted as a multivariate statistical analysis to understand the variability among different groups of peel and pulp tissues [71]. Heatmaps with cluster analysis were constructed to show the pattern of gene expressions and separate the groups of genes with similar expression.

### Gene expression using quantitative real time PCR (qRT-PCR)

The qRT-PCR was performed to validate the RNA-seq analysis on RNA extracted from peel and pulp tissues of pitaya fruits as described previously [72]. We extracted RNA from three different fruits (biological replicates) and the RNA was transcribed into cDNA using a cDNA synthesis kit (Invitrogen, Carlsbad, CA, USA). Each cDNA sample was divided into three technical replicates and qRT-PCR was performed with total nine samples. The qRT-PCR was conducted on a Roche Lightcyler® 480 instrument using the SYBR Green Master Mix (Vazyme), according to the manufacturer’s protocol. *ACT7* gene was used as an internal control. Primer sequences for target genes are present in Table S1.

## Supplementary information

**Additional file 1 **: **Table S1.** List of primers used for qRT-PCR analysis. **Table S2.** List of DEGs between RR and GW-peel. **Table S3.** List of DEGs between RR and YW-peel. **Table S4.** List of DEGs between YW and GW-peel. **Table S5.** List of DEGs between RR and GW-pulp. **Table S6.** List of DEGs between RR and YW-pulp. **Table S7.** List of DEGs between YW and GW-pulp. **Table S8**. Summary of transcription factors differentially regulated between different fruit peels and pulps. **Table S9.** Concentrations of differentially accumulated metabolites between different peel and pulp colors

## Data Availability

The datasets supporting the conclusions of this article are available in the NCBI Bioproject repository, accession number: PRJNA627818.
